# Prevalence of pulmonary tuberculosis among prison inmates: A cross-sectional survey at the Correctional and Detention Facility of Abidjan, Côte d'Ivoire

**DOI:** 10.1371/journal.pone.0181995

**Published:** 2017-07-31

**Authors:** Benjamin Séri, Ange Koffi, Christine Danel, Timothée Ouassa, Marcel-Angora Blehoué, Eric Ouattara, Jeanne-d’Arc Assemien, Jean-Marie Masumbuko, Patrick Coffie, Nathalie Cartier, Arnaud Laurent, Gilles Raguin, Denis Malvy, Thérèse N’Dri-Yoman, Serge P. Eholié, Serge K. Domoua, Xavier Anglaret, Marie-Catherine Receveur

**Affiliations:** 1 Programme PAC-CI, ANRS research site, Abidjan, Côte d’Ivoire; 2 Inserm U1219, University of Bordeaux, Bordeaux, France; 3 Centre de Diagnostic et de Recherches sur le SIDA, CHU de Treichville, Abidjan, Côte d’Ivoire; 4 Maison d’Arrêt et de Correction d’Abidjan, Abidjan, Côte d’Ivoire; 5 Interdepartmental Centre of Tropical Medicine and Clinical International Health, Division of Infectious and Tropical Diseases, Department of Medicine, University Hospital, Bordeaux, France; 6 Expertise France-Côte d’Ivoire, Abidjan, Côte d’Ivoire; 7 Département Santé, Expertise France, Paris, France; 8 Service de Gastro-entéro-hépatologie, CHU de Yopougon, Abidjan, Côte d’Ivoire; 9 Service des Maladies Infectieuses et Tropicales, CHU de Treichville, Abidjan, Côte d’Ivoire; 10 Service de Pneumologie, CHU de Treichville, Abidjan, Côte d’Ivoire; Indian Institute of Technology Delhi, INDIA

## Abstract

**Background:**

In Côte d’Ivoire, a TB prison program has been developed since 1999. This program includes offering TB screening to prisoners who show up with TB symptoms at the infirmary. Our objective was to estimate the prevalence of pulmonary TB among inmates at the Correctional and Detention Facility of Abidjan, the largest prison of Côte d’Ivoire, 16 years after this TB program was implemented.

**Methods:**

Between March and September 2015, inmates, were screened for pulmonary TB using systematic direct smear microscopy, culture and chest X-ray. All participants were also proposed HIV testing. TB was defined as either confirmed (positive culture), probable (positive microscopy and/or chest X-ray findings suggestive of TB) or possible (signs or symptoms suggestive of TB, no X-Ray or microbiological evidence). Factors associated with confirmed tuberculosis were analysed using multivariable logistic regression.

**Results:**

Among the 943 inmates screened, 88 (9.3%) met the TB case definition, including 19 (2.0%) with confirmed TB, 40 (4.2%) with probable TB and 29 (3.1%) with possible TB. Of the 19 isolated TB strains, 10 (53%) were TB drug resistant, including 7 (37%) with multi-resistance. Of the 10 patients with TB resistant strain, only one had a past history of TB treatment. HIV prevalence was 3.1% overall, and 9.6%among TB cases. Factors associated with confirmed TB were age ≥30 years (Odds Ratio 3.8; 95% CI 1.1–13.3), prolonged cough (Odds Ratio 3.6; 95% CI 1.3–9.5) and fever (Odds Ratio 2.7; 95% CI 1.0–7.5).

**Conclusion:**

In the country largest prison, pulmonary TB is still 10 (confirmed) to 44 times (confirmed, probable or possible) as frequent as in the Côte d’Ivoire general population, despite a long-time running symptom-based program of TB detection. Decreasing TB prevalence and limiting the risk of MDR may require the implementation of annual in-cell TB screening campaigns that systematically target all prison inmates.

## Introduction

The high burden of tuberculosis (TB) in African prisons is a challenging phenomenon [1–3].

It has been related to several factors, including the fact that prisoners are mostly young men originating from disadvantaged neighbourhoods with a high TB incidence, dilapidated buildings with poor inside ventilation, overcrowding, malnutrition, and, in some areas, high prevalence of human immunodeficiency virus infection (HIV) [4].

The difficulty in ensuring continued comprehensive TB care in prison leads to delay in TB diagnosis and to discontinuity in TB treatment. The latter favours the emergence of drug-resistant strains, which may further disseminate due to prison promiscuity and to the lack of facilities to perform cultures and drug sensitivity tests. After they are released, prisoners contribute to the spread of TB (including drug-resistant TB) in the communities into which they reintegrate [5,6]. Therefore, TB in prison is a public health concern for both the prisoners and the community, which makes it essential to develop comprehensive TB prison programs that include prevention, diagnosis, treatment and sentinel surveillance [7–9].

In Côte d’Ivoire, a West African country with 34 prisons harbouring more than 12,300 inmates, a TB prison program has been developed since 1999 by the national health authorities, in collaboration with MSF (Médecins Sans Frontière, 1999–2005) and ESTHER (Ensemble pour une Solidarité Thérapeutique Hospitalière En Réseau, from 2008 until today) [10]. This program includes TB case detection using the microscopic examination of acid-fast bacilli (AFB) in prisoners with TB-symptoms, first at their admission in prison and whenever they show up at the prison infirmary thereafter. Chest X-ray, TB culture and GeneXpert tests are not included in the program.

The aim of the present study was to estimate the prevalence of pulmonary TB among inmates at the Correctional and Detention Facility of Abidjan (*Maison d’Arrêt et de Correction d’Abidjan*, *MACA*), the largest prison of Côte d’Ivoire, 16 years after the prison TB program was set up.

## Methods

### Setting

MACA was built in 1980. It was completely evacuated during the 2010–2011 civil conflicts, and then gradually re-filled afterwards. In 2015, its inmate population was approximately 4,600, for an originally scheduled capacity of 1,500 prisoners. It is organized into five areas: the administration area, the women's detention area, the minors’ detention area, the men’s detention area, and the infirmary. The women area comprises only one building, as well as the minors area. The men’s area comprises four buildings: (i) a building for remand prisoners; (ii) a short-term detention building, (iii) a long-term detention building and; (iv) a detention building for personalities. The infirmary comprises three rooms: an outpatient room, an isolation room for inpatients with confirmed TB, and a room for other inpatients.

### Design and population

We conducted a systematic cross-sectional study at three buildings of MACA: the men’s long-term detention building, the women’s detention building, and the infirmary inpatients ward. In the latter, inpatients at the isolation room already treated for confirmed TB when the survey begun were excluded. All other prisoners from the three buildings who signed the informed consent were included. The reasons for choosing the three buildings were the following. We chose in priority the men’s long-term detention building, and excluded the men’s remand building, because we aimed at focusing on prisoners with long sentences, ie those with the lower turnover rate and the higher prison-related TB exposure. For women, we did not distinguish between long and short sentences, because the overall number of female prisoners at MACA is low and all are in the same building. Finally, we excluded the minors area because running a survey in this area raises specific reglementary issues.

### Clinical procedures

During a pre-survey phase that started in November 2014 and ended in February 2015, information and awareness meetings about the study were organized in each cell of the targeted buildings by the study investigators, in close collaboration with previously trained peer educators.

The survey then took place From March 1^st^ to September 30^th^ 2015. During this period, inmates were sequentially extracted from their cells by groups of 25 and taken to the infirmary outpatient room, at a pace of one group every two days. Each prisoner went through the following stages.

First, they had an individual confidential interview and medical examination by one of the study physicians. At this stage, they were asked for consent for their personal data to be anonymously recorded in the survey database, as well as for undertaking TB smear testing and HIV testing. Participants were offered the possibility to accept participating in the TB survey and refuse HIV testing.

Second, for those who accepted participation, socio-demographic, anamnestic and clinical data were recorded on a standardized form. All participants then underwent a chest X-ray and a first sputum collection, and were taken back to their cell.

Third, participants were taken back 48 hours later to the infirmary outpatient room for a second sputum collection. Those who accepted HIV testing also had a blood collection at this time.

### Biology

HIV screening was performed using the rapid test Determine HIV-1/2^®^ (Alere, Matsuhidai Matsudo Shi Chiba, Japan). HIV positivity confirmation was performed using a second rapid test, HIV-1/2 STAT-PAK^®^ (Chembio, Medford, NY).

All first and second sputum samples were microscopically examined after auramine staining, and all first sputum samples were systematically put in mycobacterial culture, irrespective of patient’s clinical and radiological data. A culture was also performed on the second sputum sample for participants who met at least one of the following criteria: (i) current cough lasting more than two weeks, (ii) abnormal chest X-ray images, (iii) HIV seropositivity, and/or (iv) positive microscopy of the first sputum sample.

Mycobacterial cultures were systematically performed on Lowenstein-Jensen (LJ) solid medium (Biorad, Marnes-la-Coquette, France) and Bactec MGIT 960 liquid medicum (Becton Dickinson Microbiology System, Sparks, NV, USA) [11,12]. Mycobacterial isolates were tested for drug susceptibility using Bactec MGIT 960 SIRE Kit (Becton Dickinson, Franklin Lakes, New Jersey, USA) [12]. The drugs tested were isoniazid, rifampicin, ethambutol and streptomycin.

The interpretation of chest X-rays was performed by two independent experiment readers.

### TB case definition

The diagnosis of TB was documented as *confirmed* (positive culture for *M*. *tuberculosis* complex), *probable* (positive microscopy with at least one acid-fast bacilli [AFB] per field, and/or chest X-ray images suggestive of TB), or *possible* [(i) at least four clinical signs or symptoms among the following: cough lasting for more than two weeks, fever lasting for more than two weeks, recent loss of appetite, chest pain, objectified body mass index lower than 18.5 Kg/m^2^ or objectified fever with body temperature ≥37.5°C on physical examination; or (ii) at least three clinical signs or symptoms *and* HIV seropositivity *or* positive microscopy with less than one AFB per field].

A mycobacterial strain was defined as pan-susceptible if it was susceptible to isoniazid, rifampicin, ethambutol and streptomycin and as multidrug-resistant if it was resistant to at least isoniazid and rifampicin.

All patients documented with TB received a TB treatment as per the National Tuberculosis Control Program guidelines [13,14].

### Statistical analysis

The association between confirmed TB and other variables was analysed using univariate and multivariate logistic regression. All variables associated with TB with a significance level ≤ 0.25 in univariate analysis were included in multivariate analysis.

Statistical analyses were carried out using the SAS 9.3 software.

Study questionnaire and data used for the analysis are available on Supporting Information (S1 Questionnaire and S1 Dataset).

### Ethics

This study was approved by the National Ethics Committee for Health and Life Sciences, the National Tuberculosis Control Program and the Ministry of Health of Côte d'Ivoire. Signed informed consent was obtained from all participants. The interviewers informed the inmates that refusal to participate would impact neither their relationship with the prison team nor their medical care.

Each participant was assigned an anonymous number. A participant register was used to ensure correspondence between the identification data (surname, forenames and date of birth) and the anonymous number of each participant. Only the study physician and coordinator had access to this register.

## Results

### Participant characteristics (Fig 1, Tables 1 and 2)

A total of 943 inmates participated in the study, including 10 from the infirmary inpatient ward, 62 from the women’s building and 871 from the men’s buildings. Participants from the two latter originated from 35 cells, including 26 cells from the men’s buildings and 9 from the women’s building. The cells had an average surface area of 44 m^2^ (standard deviation [SD] 18.5) in the men’s building and 42 m^2^ (SD 16.4) in the women’s building, an average number of 33 (SD 19) and 8 (SD 5) inmates per cell, and therefore an average available surface area of 1.3 m^2^ and 5.2 m^2^ per inmate, respectively.

**Fig 1:**
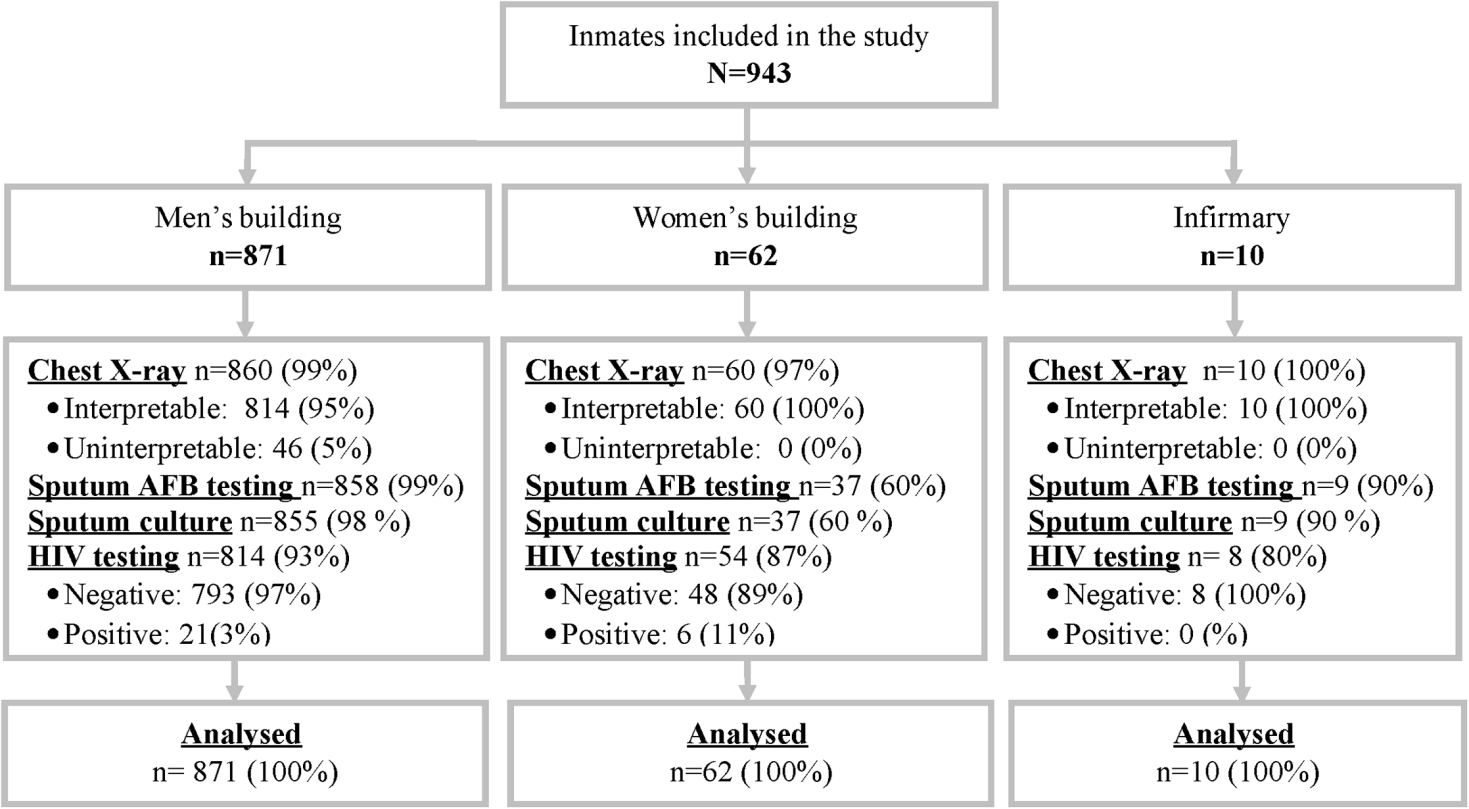
Flow chart. The inclusion criteria in the study were: (i) being inmate at the men’s long-term detention building, the women’s detention building or the infirmary inpatients ward during the study period; (ii) signing the informed consent form. 100% of participants were supposed to undergo TB tests (chest X-Ray, sputum AFB testing, sputum culture). As shown in Fig 1, in the men’s long-term detention building (92% of the overall sample size), 99% of participants actually had an X-Ray, 99% had AFB testing, and 98% had sputum culture. These figures were 97%, 60% and 60% in the women’s building (7% of the overall sample size). The reasons why these figures are not 100% are logistical (some participants could not provide sputum; some X-Rays failed for technical reasons). Finally, all participants were proposed HIV testing, but only those who accepted underwent the test.

**Table 1:** Socio-demographic and clinical characteristics.

	Total (N = 943)	Men’s building (N = 871)	Women’s building (N = 62)	Infirmary (N = 10)
**Past history of imprisonment, n (%)**				
No	597 (83)	589 (83)	7 (88)	1 (50)
Yes	119 (17)	117 (17)	1 (12)	1 (50)
**Term of current imprisonment, median (IQR), months**	22 (11–32)	23 (13–33)	5 (1–14)	21 (16–28)
**Term of current imprisonment, n (%)**				
< 3 months	56 (6)	35 (4)	21 (34)	-
3–6 months	72 (8)	58 (7)	14 (23)	-
6–12 months	126 (13)	115 (13)	10 (16)	1 (12)
1 to 2 years	272 (29)	256 (29)	12 (20)	4 (44)
More than 2 years	415 (44)	407 (47)	4 (7)	4 (44)
**Median age, (IQR), years**	31 (26–37)	31 (26–37)	29 (24–39)	32 (29–40)
**Nationality, n (%)**				
Ivoirian	742 (79)	681 (78)	52 (84)	9 (90)
Other	201 (21)	190 (22)	10 (16)	1 (10)
**Education level, n (%)**				
Never schooled	271 (29)	247 (28)	22 (36)	2 (20)
Primary	272 (29)	258 (30)	12 (19)	2 (20)
Secondary and above	400 (42)	366 (42)	28 (45)	6 (60)
**Previous place of residence, n (%)**				
Abidjan	795 (84)	730 (84)	57 (92)	8 (80)
Outside Abidjan	148 (16)	141 (16)	5 (8)	2 (20)
**Marital status, n (%)**				
Does not live with a partner	465 (49)	427 (49)	30 (48)	8 (80)
Lives with a partner	478 (51)	444 (51)	32 (52)	2 (20)
**Number of daily meals, n (%)**				
Only one meal	495 (52)	478 (55)	15 (24)	2 (20)
Two meals	348 (37)	313 (36)	31 (50)	4 (40)
At least three meals	100 (11)	80 (9)	16 (26)	4 (40)
**Origin of food, n (%)**				
Prison food only	291 (31)	284 (32)	3 (5)	4 (40)
Non-prison food only	471 (50)	407 (47)	59 (95)	5 (50)
Both	181 (19)	180 (21)	-	1 (10)
**Previous history of tuberculosis**				
No	897 (95)	827 (95)	61 (98)	9 (90)
Yes	46 (5)	44 (5)	1 (2)	1 (10)
**Smoking status, n (%)**				
Non-smoker	372 (40)	318 (37)	47 (76)	7 (70)
Ex-smoker	51 (5)	48 (5)	2 (3)	1 (10)
Active smoker	520 (55)	505 (58)	13 (21)	2 (20)
**HIV status upon admission to prison, n (%)**				
Negative	550 (59)	493 (57)	51 (82)	6 (60)
Positive	12 (1)	8 (1)	4 (7)	-
Not done	369 (40)	358 (42)	7 (11)	4 (40)

n = number; % = percentage; SD = Standard Deviation; IQR = Interquartile Range

Missing values = 227 for Past history of imprisonment, 2 for Term of current imprisonment, 4 for age and 12 for HIV status upon admission to prison.

**Table 2:** Symptoms, signs and test results.

	Total (N = 943)	Men’s building (n = 871)	Women’s building (n = 62)	Infirmary (n = 10)
**Cough > 2 weeks, n (%)**				
No	736 (78)	679 (78)	55 (89)	2 (20)
Yes	207 (22)	192 (22)	7 (11)	8 (80)
**Fever > 2 weeks, n (%)**				
No	776 (82)	722 (83)	50 (81)	4 (40)
Yes	167 (13)	149 (17)	12 (19)	6 (60)
**Chest pain, n (%)**				
No	696 (74)	641 (74)	52 (84)	3 (30)
Yes	247 (26)	230 (26)	10 (16)	7 (70)
**Recent loss of appetite, n (%)**				
No	798 (85)	744 (85)	49 (79)	5 (50)
Yes	145 (15)	127 (15)	13 (21)	5 (50)
**Body mass index, n (%), Kg/m**^**2**^				
≥ 18.5	836 (89)	771 (89)	59 (95)	6 (60)
< 18.5	107 (11)	100 (11)	3 (5)	4 (40)
**Objectified Temperature, n (%)**				
≤ 37°C	929 (99)	861 (99)	59 (95)	9 (90)
˃ 37°C	14 (1)	10 (1)	3 (5)	1 (10)
**Chest X-ray, n (%)**				
Uninterpretable	46 (5)	46 (5)	-	-
Interpretable	884 (95)	814 (95)	60 (100)	10 (100)
*Non suggestive of TB*	*841 (95)*	*777 (95)*	*59 (98)*	*5 (50)*
Suggestive of TB*	*43 (5)*	*37 (5)*	*1 (2)*	*5 (50)*
**Microscopy and culture, n (%)**				
Negative AFB and negative culture	766 (85)	733 (85)	29 (78)	4 (44)
Negative AFB and positive culture	11 (1)	11 (1)	-	-
Positive AFB and positive culture	8 (1)	7 (1)	-	1 (12)
Positive AFB and negative culture	119 (13)	107 (17)	8 (22)	4 (44)
*< 1 AFB/ field*	*115 (97)*	*104 (97)*	*8 (100)*	*3 (75)*
*≥ 1 AFB/field*	*4 (3)*	*3 (3)*	*-*	*1 (25)*
**HIV serology, n (%)**				
Not performed	67 (7)	57 (6)	8 (13)	2 (20)
Performed	876 (93)	814 (94)	54 (87)	8 (80)
*Negative*	*849 (96*.*9)*	*793 (97*.*4)*	*48 (88*.*9)*	*8 (100)*
*Positive*	*27 (3*.*1)*	*21 (2*.*6)*	*(11*.*1)*	*-*

n = number; % = percentage; Kg/m^2^ = Kilogram per square meter

*excavations (n = 2), alveolar opacities in the apex (n = 11), alveolar opacities in other settings (n = 25) and pleural effusions (n = 5).

Missing values = 13 for Chest X-ray and 39 for Microscopy and culture.

Overall, the study participants had a median age of 31 years (IQR: 26–37) [31 years (IQR: 26–37) in the men’s building, 29 years (IQR: 24–39) in the women’s building and 32 years (IQR: 29–40) among infirmary inpatient], and had been in detention for a median of 22 months (IQR: 11–32) [23 months (IQR: 13–33) in the men’s building, 5 months (IQR: 1–14) in the women’s building and 22 months (IQR: 16–28) among infirmary inpatient]. Five percent had a past history of TB, 22% declared a cough lasting for more than two weeks, 13% a fever lasting for more than two weeks, 15% a recent loss of appetite, and 26% chest pains. On physical examination, 11% were found with a body mass index lower than 18.5 Kg/m^2^ and 1% had objectified fever.

### Tests (Table 2)

Chest X-rays were performed in 930 participants (99%). Forty-six (5%) were deemed not interpretable, all of them from inmates at the men’s building. Of the remaining 884, 43 (5%) were considered suggestive of TB, on the basis of excavations (n = 2), alveolar opacities in the apex (n = 11), alveolar opacities in other settings (n = 25) and pleural effusions (n = 5). Of note, 13 out of these 43 participants had declared a past history of pulmonary TB.

A total of 1821 sputum specimens were collected in 904 participants (96%). Of these 1821 specimens, 1272 had AFB testing and 1251 were cultured. Five hundred and thirty-six inmates (59%) had one AFB testing, 368 (41%) had two AFB testings, 551 (61%) had one sputum culture, and 350 (39%) had two cultures. Overall, 19 inmates had at least one positive culture (including 8 with positive AFB smear), and 119 had at least one positive AFB smear with a negative culture or a culture non available. Of the latter, 115 (97%) had less than one AFB per field.

### TB cases (Tables 3 and 4)

Overall, 88 (9.3%) participants met the TB case definition, including 19 (2.0%) with confirmed TB, 40 (4.2%) with probable TB and 29 (3.1%) with possible TB. Of the 88 inmates found with TB, 84% had been in prison more than 12 months.

**Table 3:** Tuberculosis cases.

	Total (N = 943)	Men’s building (N = 871)	Women’s building (N = 62)	Infirmary (N = 10)
**Overall TB cases, n (%)**	**88 (9.3)**	**78 (9.0)**	**3 (4.8)**	**7 (70.0)**
Possible TB ^(1)^	29 (3.1)	26 (3.0)	2 (3.2)	1 (10)
Probable TB ^(2)^	40 (4.2)	34 (3.9)	1 (1.6)	5 (50)
Confirmed TB ^(3)^	19 (2.0)	18 (2.1)	-	1 (10)
Pan-susceptible	9 (47)	8 (44)	-	1 (100)
Resistant	10 (53)	10 (55)	-	0
Multidrug-resistant	*7 (37)*	*7 (39)*	*-*	0
Other resistance ^(4)^	*3 (16)*	*3 (16)*	*-*	0

n = number; % = percentage

^(1)^ *Confirmed* TB: positive culture for *M*. *tuberculosis* complex

^(2)^ *Probable* TB: negative culture or culture unavailable, positive microscopy with at least one acid-fast bacillus [AFB] per field, and/or chest X-ray images highly suggestive of TB

^(3)^ *Possible*: TB (i) at least four clinical signs or symptoms suggestive of TB, or (ii) at least three clinical signs or symptoms suggestive of TB *and* HIV seropositive *or* positive microscopy with less than one AFB per field

^(4)^ Isoniazid mono-resistance, n = 1; Isoniazid and ethambutol resistance, n = 2

**Table 4:** Tuberculosis documentation.

	N	X-Ray images suggestive of TB	AFB positive		Culture positive	Number of symptoms*					
			< 1 AFB	≥ 1 AFB		0	1	2	3	4	5
**TB cases**											
** Confirmed**	19	7	5	3	19	3	6	5	2	1	2
** Probable**	40	36	14	4	0	12	15	5	3	4	1
** Possible**	29	0	10	0	0	0	0	0	8	18	3
**Non TB cases**	855	0	91	0	0	441	222	129	61	2	0

AFB: acid fast bacilli

TB: tuberculosis

* among the following ones: cough lasting for more than two weeks, fever lasting for more than two weeks, recent loss of appetite, chest pain, objectified body mass index lower than 18.5 Kg/m^2^

Of the 19 cases of confirmed TB, 18 were found in the men’s building and one among the infirmary inpatients. Three out of these 19 patients had none of the sign or symptoms listed in Table 2. Seven out of these 19 patients had X-Ray images suggestive of TB.

All 19 isolated TB strains were tested for resistance, of which 10 (53%) were found resistant, including 7 (37%) with multi-resistance. Of the 10 patients with TB resistant strain, only one had a past history of TB treatment.

Of the 69 cases of probable or possible TB, 60 were found in the men’s building, 3 in the women’s building and 6 among the infirmary inpatients. Of these 69 participants, 55% had a cough lasting for more than two weeks and 61% had chest pains; 49% self-reported fever lasting for more than two weeks and 45% loss of appetite; 30% had a body mass index lower than 18.5 Kg/m^2^; and 57 (83%) had at least one of these signs or symptoms. In addition, 36 (52%) had X-Ray images suggestive of TB.

HIV testing was performed in 876 participants (93%), of whom 27 (3.1%) were positive. (men 2.6%, women 11.1%); including in 83 participants with TB, of whom 8 (9.6%) were positive (TB confirmed cases 5.6%, TB probable cases 2.7%; TB possible cases 21.4%).

### Factors associated with TB (Table 5)

The analysis of factors associated with confirmed TB was restricted to participants from the men’s building. In multivariate analysis, factors significantly associated with TB were age ≥30 years (Odds Ratio 3.8; 95% CI 1.1–13.3), cough (OR 3.6; 95% CI 1.3–9.5) and self-reported fever (OR 2.7; 95% CI 1.0–7.5).

**Table 5:** Factors associated with confirmed tuberculosis.

		Overall population N	Confirmed TB n (%)	Univariate analysis			Multivariate analysis**			
				OR	95%CI	*p*	OR	95%CI	*p*
Previous prison stay*	No	558	9 (1.6%)	ref	-	**0.41**	-	-	**-**
	Yes	108	3 (2.8%)	1.7	0.5–6.5		-	-	**-**
Previous history of tuberculosis*	No	778	16 (2.1%)	ref		**0.15**	-	-	**-**
	Yes	33	2 (6.1%)	3.1	0.7–13.9		-	-	**-**
Previous residence*	Abidjan	679	12 (1.8%)	ref	-	**0.06**	-	-	**-**
	Other	132	6 (4.5%)	2.6	1.0–7.2		-	-	**-**
Previous stable partnership*	No	396	9 (2.3%)	ref	-	**0.92**	-	-	**-**
	Yes	415	9 (2.2%)	0.9	0.4–2.4		-	-	**-**
**Age**	< 30 years	342	3 (0.9%)	ref		**0.04**	ref	-	**0.04**
	> 30 years	467	15 (3.2%)	3.8	1.1–13.1		3.8	1.1–13.3	
**Nationality**	Ivoirian	642	12 (1.9%)	ref	-	**0.19**	-	-	**-**
	Other	169	6 (3.6%)	1.9	0.7–5.2		-	-	**-**
**Schooling**	Never Schooled	228	8 (3.5%)	2.0	0.7–6.0	**0.31**	-	-	**-**
	Primary	240	4 (1.7%)	0.9	0.3–3.4		-	-	**-**
	Secondary	343	6 (1.7%)	ref	-		-	-	**-**
**Time since incarceration**	≤ 1 year	202	4 (2.0%)	ref	-	**0.94**	-	-	**-**
	1–2 years	234	5 (2.1%)	1.1	0.3–4.1		-	-	**-**
	> 2 years	375	9 (2.4%)	1.2	0.4–4.0		-	-	**-**
**Number of daily meals**	One	438	12 (2.7%)	2.1	0.3–16.5	**0.55**	-	-	**-**
	Two	297	5 (1.7%)	1.3	0.1–11.2		-	-	**-**
	Three	76	1 (1.3%)	ref	-		-	-	**-**
**Origin of food**	Prison food	255	8 (3.1%)	3.1	0.9–10.3	**0.06**	-	-	**-**
	Non-prison food	385	4 (1.0%)	ref	-		-	-	**-**
	Both	171	6 (3.5%)	3.5	1.0–12.4		-	-	**-**
**Smoking status**	Never smoked	306	6 (2.0%)	ref	-	**0.93**	-	-	**-**
	Ex-smoker	44	1 (2.3%)	1.1	0.1–9.9		-	-	**-**
	Active smoker	461	11 (2.4%)	1.2	0.4–3.3		-	-	**-**
**Recent loss of appetite**	No	710	14 (2.0%)	ref	-	**0.21**	-	-	**-**
	Yes	101	4 (4.0%)	2.1	0.7–6.4		-	-	**-**
**Chest pain**	No	617	10 (1.6%)	ref	-	**0.05**	-	-	**-**
	Yes	194	8 (4.1%)	2.6	1.1–6.7		-	-	**-**
**Cough > 2 weeks**	No	649	9 (1.4%)	ref	-	**0.003**	ref	-	**0.01**
	Yes	162	9 (5.6%)	4.2	1.6–10.7		3.6	1.3–9.5	
**Fever > 2 weeks**	No	691	11 (1.6%)	ref	-	**0.007**	ref	-	**0.05**
	Yes	120	7 (5.8%)	3.8	1.4–10.1		2.7	1.1–7.5	
**Body mass index**	≥ 18.5 Kg/m^2^	730	15 (2.1%)	ref	-	**0.35**	-	-	**-**
	< 18.5 Kg/m^2^	81	3 (3.7%)	1.8	0.5–6.5		-	-	**-**
**HIV serology**	Negative	741	16 (2.2%)	ref	-	**0.30**	-	-	-
	Positive	16	1 (6.3%)	3.0	0.4–24.3		-	-	-

* Prior to current incarceration

** Final model

TB: tuberculosis; n = number; % = row percentage; OR = Odds Ratio; CI = Confidence Interval

SD = Standard Deviation; Kg/m2 = Kilogram per square meter; ref: reference

N = overall population: 811 for all variables, except those with missing values. Missing values = 145 for Previous prison stay, 2 for Age and 54 for HIV serology

## Discussion

Our study demonstrates the high burden of TB at MACA, the largest prison in Côte d’Ivoire.

TB prevalence was very high, between 2.0%, a conservative estimate restricted to culture-confirmed cases, and 9.3%, an estimate including non culture-confirmed cases. These figures are 10 to 44 folds as high as the Côte d’Ivoire national TB prevalence, recently estimated at 0.23% [15]. In addition, 53% of isolated strains were resistant to at least one TB drug, including 37% that were multi-drug resistant.

Overcrowding is a well known condition for TB transmission in prison [16–18]. The average living surface area at MACA is 1.3 m^2^ per male inmate, well below the 4m^2^ recommended by the WHO [19].

Our estimated TB prevalence falls within the range of figures found in other prison settings across the African continent [17,18,20–27]. Importantly, though, this high prevalence was found after 16 years of a program that made TB screening and treatment routinely available in the main prisons of Côte d’Ivoire. This TB program consists of offering symptom-driven TB screening, first on prison admission, and then whenever prisoners show up with symptoms at the infirmary. However, it does target neither the inmates who do not self-declared symptoms, nor those who have symptoms but cannot make it to the infirmary.

As expected, TB was frequent among inmates seeking care at the infirmary. Under ongoing procedures, the seven TB cases diagnosed at the infirmary during the study would have been diagnosed and treated if our study had not been done. However, these cases represented only 8% of the overall TB cases found during the study. The remaining 92% were diagnosed thanks to a systematic in-cell screening of all inmates, and would likely have long remained undiagnosed outside the context of this study [18,28].

Screening individuals who make it to the infirmary leads to restrict TB diagnosis to inmates who can access the prison health services, a tricky condition that partly depends on inmates own internal rules [17,18,21,27,29–33]. Furthermore, symptoms-based TB screening algorithms may have low performance in prison context [25]. Our data thus consistently reinforce the suggestion that a systematic in-cell screening should be recurrently proposed to all prisoners, in order for TB cases to be diagnosed more efficiently and treated more rapidly.

Even if based on a limited number of culture-proven cases, our 37% MDR rate appears to be in the higher range of figures reported in other African prisons [22,31]. It is also much higher than the overall rate of MDR in Côte d’Ivoire, currently estimated at 3% among new TB cases and 14% among recurrent cases [34]. Only 5% of our study population self-declared a previous episode of TB. Our high rate of MDR TB is unlikely to be explained by a high number of individuals under-declaring a past history of TB, and more likely to have two other explanations. First, the risk of TB transmission inherent to prison conditions increases the risk for MDR strains to be primarily transmitted [16,35]. Second, the difficulty in getting access to health care facilities while being in prison increases the risk of poor adherence to treatment and of developing secondary resistance to TB drugs [36]. Whereas the former emphasizes the need for a more systematic TB screening as highlighted above, the latter suggests the need for improving adherence to TB treatments in prison. Fighting against TB drug resistance inside the prison is not only of utmost importance for prisoners: it is also important for the outside community, as prisoners with untreated MDR TB will disseminate it once released [36,37].

As expected, cough and fever lasting more than two weeks were significantly associated with the risk of confirmed TB [26,38,39]. Other classical factors such as HIV seropositivity [18,25,30,40,41], low body mass index [17,18,25], active smoking [38,42] and chest pain [25] were not significantly associated with TB, due to the lack of power resulting from the relatively low absolute number of confirmed TB cases. The prevalence of HIV infection was comparable to that in the general adult population of Côte d’Ivoire [43].

The strengths of our study are the fact that all inmates, not only those with symptoms and not only those who showed up at the infirmary, were screened for TB; and that we systematically used TB culture. Both procedures allow us for a robust lower bound estimate for TB prevalence. In our study, however, we also used a combination of clinical, AFB screening and radiological criteria to document non-culture proven TB. Each of these tests considered separately have their own limitations, and taken together they provide us with a higher bound estimate for TB prevalence that should be considered with caution. Chest X-ray interpretation is a function of X-ray quality and readers experience, and has low sensitivity [40]. Sputum-smear microscopy sensitivity is much lower than that of GeneXpert, a test that we did not use [44]. Screening systematically all prison inmates is a complex logistical process that do not allow repeating tests as much as it may be required. In our study, only 41% of inmates had two AFB testing, 96% had at least one culture and 37% had two cultures. Whereas it may be suggested that systematic TB screening campaign in prison should use GeneXpert rather than Sputum-smear microscopy, the question of whether the latter should be combined with other tests remains open.

The main study target was TB, because we made the hypothesis that it was an overlooked threatening disease at MACA, and that estimating the importance of this problem would encourage the country authorities to implement useful measures against it. However, TB is not the only problem to deal with, and that further studies should focus on sexually transmitted diseases [45]. Finally, we were unable to have a genotypic study of the 19 isolates, which could have illustrated to which extend TB transmission occurs inside the prison. Although the high prevalence of TB at MACA compared to figures in the general population clearly suggests that there is TB transmission ongoing inside, it does not prove it.

In conclusion, our data suggest that the current program using passive symptoms-based screening is not sufficient to decrease the risk of TB and of TB drug resistance in the main prison of Côte d’Ivoire. Decreasing TB prevalence and limiting the risk of MDR may require the implementation of annual TB screening campaigns that would systematically target all prison inmates. The best TB tests or combination of tests to be used in such campaigns, ie those that would have be the most cost-effective and be routinely feasible, still remain to be identified.

## Supporting information

S1 Questionnaire Questionnaire used for data collection. (PDF)

S1 Dataset Datasets used for the manuscript. (XLS)
