# Supplementary material for: Prevalence of pulmonary tuberculosis among prison inmates: A cross-sectional survey at the Correctional and Detention Facility of Abidjan, Côte d'Ivoire
Source: PLoS One. 2017 Jul 31;12(7):e0181995. doi: 10.1371/journal.pone.0181995 (PMC5536365; doi:10.1371/journal.pone.0181995)
Supplement: S1 Questionnaire — (PDF) [file pone.0181995.s001.pdf]

Study number |__||__| |__||__||__||__| Warrant of committal: |__||__|/|__||__|/|__||__||__||__|

<table>
<tr><td>Q 0.1</td><td colspan="2">Date of inclusion: |__||__| / |__||__| / |__||__| |__||__|</td><td></td></tr>
<tr><td>Q 0.2</td><td colspan="2">Building: ❑ Building C 1 ❑ Women's building 2 ❑ CAT 3
❑ Infirmary 4 ❑ Other building 5</td><td>|__|</td></tr>
<tr><td>Q 0.3</td><td colspan="2">Floor:</td><td>|__|</td></tr>
<tr><td>Q 0.4</td><td colspan="2">Cell:</td><td>|__||__|</td></tr>
<tr><td>Q 0.5</td><td colspan="2">Number of inmates per cell: |__||__||__| ❑ Does not know 99</td><td></td></tr>
<tr><th colspan="4">1- SOCIO-DEMOGRAPHIC CHARACTERISTICS</th></tr>
<tr><td>Q 1.1</td><td colspan="2">Gender: ❑ Male 1 ❑ Female 2</td><td>|__|</td></tr>
<tr><td>Q 1.2</td><td colspan="2">Date of birth: |__||__| / |__||__| / |__||__| |__||__| Or Age: |__||__| years
❑ Does not know 99</td><td></td></tr>
<tr><td>Q 1.3</td><td colspan="2">Area of residence before entering MACA:
❑ Abobo1 ❑ Adjamé / Williamsville 2 ❑ Attécoubé/Lokodjoro3 ❑ Cocody/Deux Plateau 4
❑ Koumassi 5 ❑ Marcory6 ❑ Plateau7 ❑ Port-Bouët/Vridi8 ❑ Treichville 9 ❑ Yopougon 10
❑ Bingerville11 ❑ Anyama12 ❑ Grand Bassam13 ❑ Autre14
If other, specify: ____________________</td><td>|__|__|</td></tr>
<tr><td>Q 1.4</td><td colspan="2">Nationality: ❑ Ivorian 1 ❑ Other 2
If other, specify: ____________________</td><td>|__|</td></tr>
<tr><td>Q 1.5</td><td colspan="2">Education level:
❑ Unschooled 1 ❑ Primary 2 ❑ Secondary 3 ❑ Higher 4</td><td>|__|</td></tr>
<tr><td>Q 1.6</td><td colspan="2">Current marital status:
❑ Single 1 ❑ Monogamous marriage 2 ❑ Polygamous marriage 3 ❑ Cohabiting 4
❑ Divorced 5 ❑ Widow(er) 6</td><td>|__|</td></tr>
<tr><td>Q 1.7</td><td colspan="2">HIV status upon entering prison:
❑ Positive 1 ❑ Negative 2 ❑ Indeterminate 3 ❑ Not done 4</td><td>|__|</td></tr>
<tr><td>Q 1.8</td><td colspan="2">If HIV status known, date of HIV test : |__||__| / |__||__| / |__||__||__||__|
❑ Does not know 99</td><td></td></tr>
<tr><td>Q 1.9</td><td colspan="2">How many meals do you regularly eat per day?
❑ No meal 1 ❑ Only one meal 2 ❑ Two meals 3 ❑ Three meals 4 ❑ More than three meals 5</td><td>|__|</td></tr>
<tr><td>Q 1.10</td><td colspan="2">Where do the meals you eat in your cell mostly come from?
(Many answers possible)
❑ Only the prison food ❑ I cook my own food
❑ Group meals among fellow inmates ❑ Other
If other, specify: ____________________</td><td></td></tr>
<tr><td>Q 1.11</td><td colspan="2">Where does the water you drink in your cell mostly come from?
❑ Tap water 1 ❑ Mineral water 2 ❑ Packaged water of unknown origin 3 ❑ Other 4
If other, specify: ____________________</td><td>|__|</td></tr>
<tr><th colspan="4">2 – PAST MEDICAL HISTORY</th></tr>
<tr><td>Q 2.1</td><td colspan="2">In the past, did you have a disease requiring a treatment for many months?
❑ Yes 1 ❑ No 0</td><td>|__|</td></tr>
<tr><td colspan="2">Q2. 1 A</td><td>If yes, was it a pulmonary infection? ❑ Yes 1 ❑ No 0</td><td>|__|</td></tr>
<tr><td colspan="2">Q2. 1 B</td><td>Is yes, which one? ____________________</td><td></td></tr>
<tr><td>Q 2.2</td><td colspan="2">Since you are in prison, have you heard that another inmate in your cell has Tuberculosis?
❑ Yes 1 ❑ No 0</td><td>|__|</td></tr>
<tr><td>Q 2.3</td><td colspan="2">Have you ever had tuberculosis? ❑ Yes 1 ❑ No 0</td><td>|__|</td></tr>
<tr><td></td><td>Q 2.3 A</td><td>If yes, TB treatment start date:
|__||__| / |__||__| / |__||__| |__||__| ❑ Does not know 99</td><td></td></tr>
</table>

	Q 2.3 B	**Treatment duration:** |__||__| **months** ❑ **Does not know** 99	
Q 2.4	**Do you have asthma?** ❑ Yes 1 ❑ No 0		|__|
Q 2.5	**Do you have diabetes?** ❑ Yes 1 ❑ No 0		|__|
	Q 2.5 A	**If yes, are you currently on anti-diabetic therapy?** ❑ Yes 1 ❑ No 0	|__|
	Q 2.5 B	**If currently on anti-diabetic therapy, specify the type of therapy:** ______	
Q 2.6	**Have you ever smoked tobacco?** ❑ No 1 ❑ Yes, in the past 2 ❑ Yes, currently 3		|__|
	Q 2.6 A	**If currently smoking, how often did you smoke tobacco in the last 30 days?** ❑ Only 1 to 3 times 1 ❑ 1 to 3 times a week 2 ❑ 4 to 6 times a week 3 ❑ Once a day 4 ❑ 2 to 3 times a day 5 ❑ 4 times a day 6	|__|
	Q 2.6B	**How many cigarettes do you smoke per day?** |__||__||__| cigarettes	|__||__||__|
Q 2.7	**Is it your first imprisonment?** ❑ Yes 1 ❑ No 0		|__|
	Q 2.7 A	**If "no", what was the length of your previous imprisonment?** ❑ Less than 3 months 1 ❑ 3 to 6 months 2 ❑ 6 to 12 months 3 ❑ 1 to 2 years 4 ❑ More than 2 years 5	|__|

SURVEY FORM-TO BE COMPLETED BY- THE PHYSICIAN

**3- CLINICAL CHARACTERISTICS**			
Q 3.1	**Weight: |__||__||__| kg**		|__||__||__|
Q 3.2	**Height: |__||__||__| cm**		|__||__||__|
Q 3.3	**Temperature: |__||__|, |__| degree**		|__||__|,|__|
Q 3.4	**Symptoms:**		
	Q 3.4.1	**Cough more than 2 weeks ago or cough in the last month?** ❑ Yes 1 ❑ No 0	|__|
	Q 3.4.2	**Weight loss above 10% of body weight?** ❑ Yes 1 ❑ No 0	|__|
	Q 3.4.3	**Recent loss of appetite:** ❑ Yes 1 ❑ No 0	|__|
	Q 3.4.4	**Chest pain:** ❑ Yes 1 ❑ No 0	|__|
	Q 3.4.5	**Fever more than 2 weeks ago:** ❑ Yes 1 ❑ No 0	|__|
**4- Decision after performing chest X-ray and HIV test**			
Q 4.1	**At least one clinical symptom:** ❑ Yes 1 ❑ No 0		|__|
Q 4.2	**Positive HIV test or past medical history:** ❑ Yes 1 ❑ No 0		|__|
Q 4.3	**Chest X-ray:** ❑ Abnormal 1 ❑ Normal 0		|__|
Q 4.4	**Positive D1 sputum** ❑ Yes 1 ❑ No 0		|__|
In case of positive answer to at least one of the previous questions, patient sputum samples should be sent to CeDReS for direct examination and culture			
Q 4.5	**Have sputum samples been sent to CeDReS?** ❑ Yes 1 ❑ No 0		|__|
	Q 4.5 A	**If yes, date of submission:** |__||__| / |__||__| / |__||__||__||__|	
	Q 4.5 B	**If no, specify the reasons:** ______	

Investigator's name and signature:

Physician's name and signature:
